# Endoscopic Kehr’s T-Tube Placement to Treat Persistent Large Gastro-cutaneous Fistula After One Anastomosis Gastric Bypass: Video Demonstration

**DOI:** 10.1007/s11695-022-06285-5

**Published:** 2022-09-22

**Authors:** Arnaud Liagre, Michel Queralto, Jean Marc Combis, Paulo Peireira, Jane N. Buchwald, Francesco Martini, Niccolo Petrucciani

**Affiliations:** 1 Clinique Des Cedres, Bariatric Surgery Unit, Ramsay Générale de Santé, Cornebarrieu, France; 2 Clinique Des Cedres, Gastrointestinal Endoscopy Unit, Ramsay Générale de Santé, Cornebarrieu, France; 3 Clinique A. Paré, Gastrointestinal Endoscopy Unit, Toulouse, France; 4 Clinique A. Paré, Bariatric Surgery Unit, Toulouse, France; 5 Division of Scientific Research Writing, Medwrite Medical Communications, Maiden Rock, WI USA; 6 Department of Medical and Surgical Sciences and Translational Medicine, Faculty of Medicine and Psychology, St Andrea Hospital, Sapienza University, via di Grottarossa 1035-9, 00139 Rome, Italy

**Keywords:** One Anastomosis Gastric Bypass, Fistula, Video, Endoscopy, T-Tube

## Abstract

**Background:**

We aim to show the endoscopic placement of a T-tube to treat a persistent large gastro-cutaneous fistula after OAGB.

**Methods:**

We present the case of a 46-year-old woman with BMI of 48 kg/m^2^, who underwent OAGB and was re-operated on the 2nd postoperative day (POD) for leakage. Washing and drainage of the abdominal cavity was performed, and no fistulous orifice was identified. An upper gastrointestinal (GI) endoscopy was performed at POD 20 for the persistence of leakage of 150 ml/day by the drain and a gastric fistulous orifice of 2 cm was detected.

**Results:**

At POD 22, under general anesthesia, upper GI endoscopy was performed and a T-tube was placed in the fistulous orifice with a “rendez-vous” technique (as demonstrated in the Video), placing the T branch in the digestive lumen pressed against the wall and the long part of the T exiting at the cutaneous orifice. The T-tube was clamped after 3 days and the patient could be gradually re-fed. The patient was discharged 8 days after the procedure, with perfect clinical tolerance and no complications. The ablation of the tube one was performed on POD 84. No relapse occurred during a follow-up of 48 months.

**Conclusion:**

Persistent large gastro-cutaneous fistulas with an orifice bigger than 1 cm in diameter are difficult to manage. The endoscopic placement of a T-tube seems a useful option, which may facilitate the healing of the fistula. Further studies are needed to better define the role of this procedure.

**Supplementary Information:**

The online version contains supplementary material available at 10.1007/s11695-022-06285-5.

## Introduction

Persistent large gastro-cutaneous fistulas after one anastomosis gastric bypass (OAGB) are difficult to treat. Endoscopic T-tube placement has been described with promising results [2]. In this article, we aim to show the endoscopic placement of a T-tube to treat a large gastric fistula after OAGB.

## Materials and Methods

We present the case of a 46-year-old woman with a BMI of 48 kg/m^2^, who underwent OAGB and was re-operated on the 2nd postoperative day (POD) for severe sepsis. During the re-laparoscopy at POD 2, washing and drainage of the abdominal cavity was performed, and no fistulous orifice was identified. An upper gastrointestinal (GI) endoscopy was performed at POD 20 for the persistence of leakage of 150 ml/per day by the drain and a gastric fistulous orifice of 2 cm was detected. Reference endoscopic treatments were judged not useful in this case (Pigtail was not suitable because the orifice was too large, a stent because of OAGB). The placement of a T-tube by endoscopy was proposed. The placement of the T-tube by laparoscopy is part of the therapeutic arsenal in case of fistulas after OAGB [1]. We have extended its use to the endoscopic route to reduce the invasiveness of the procedure [2].

## Results

The patient was supine under general anesthesia. An upper GI endoscopy was performed at POD 22, objectifying a wide gastric fistula orifice. The “rendez-vous” technique (demonstrated in the Video) for T-tube placement included: (1) placement of a Boston Scientific Jagwire® guide wire along the drain path from the stomach to the skin orifice via the fistula; (2) removal of the drain while maintaining the guide wire; (3) fixation of an Exacto® Steris diathermic loop to the guide wire at the cutaneous orifice and progression of the loop in the opposite direction from the guide wire to the mouth; (4) fixation of a T-tube® Coloplast 14 French (long part of the T) to the loop and progression of the T-tube in the opposite direction to have the T branch in the digestive lumen pressed against the wall and the long part of the T exiting at the cutaneous orifice; and (5) fixation of the T-tube at the skin. After the procedure, no complications occurred. The T-tube was clamped 3 days after the endoscopic procedure (at POD 25) and the patient could be gradually re-fed. A purulent leak around the T-tube persisted for a few days requiring local nursing care. The patient was discharged 8 days after the procedure (POD 30). The clinical tolerance of the T-tube was perfect. The ablation of this one was carried out on POD 84 in the outpatient clinic. No relapse occurred during a follow-up of 48 months.

## Conclusion

Persistent large gastro-cutaneous fistulas with an orifice of more than 1 cm in diameter are difficult to manage. The endoscopic placement of a T-tube seems a useful option, which may facilitate the healing of the fistula. It is based on the intense inflammatory reaction around the latex T-tube. Further studies are needed to better define the role of this procedure.

## Supplementary Information

Below is the link to the electronic supplementary material.

Supplementary file1 (MP4 82075 KB)
